# Mantle cell lymphoma presenting with severe upper gastrointestinal bleeding: A case report

**DOI:** 10.1097/MD.0000000000029236

**Published:** 2022-06-10

**Authors:** Khaled Ali, Mhd Baraa Habib, Ferial Alloush, Mohamed A. Yassin

**Affiliations:** a Community Medicine Department, Hamad Medical Corporation, Doha, Qatar; b Department of Internal Medicine, Hamad Medical Corporation, Doha, Qatar; c Pathology Department, Hamad Medical Corporation, Doha, Qatar; d Hematology Department, Hamad Medical Corporation, Doha, Qatar.

**Keywords:** gastrointestinal bleeding, mantle cell lymphoma

## Abstract

**Introduction:**

Although it usually involves extranodal sites such as the gastrointestinal tract in more than 80% of cases, mantle cell lymphoma is considered a rare cause of gastrointestinal bleeding, especially severe and life-threatening bleeding.

**Patient concern:**

A 60-year-old man with peptic ulcer disease, who presented with severe upper gastrointestinal (GI) bleeding and large gastric ulcer.

**Diagnosis:**

Primary gastric mantle cell lymphoma.

**Interventions:**

He was treated conservatively with blood transfusion and started on Traneximic acid for 3 days. Then, the patient underwent urgent hemostatic radiotherapy.

**Outcomes:**

The patient became stable and kept in the hospital for monitoring with a definite diagnosis of stage IV Mantle cell lymphoma is made.

**Conclusion:**

Mantle cell lymphoma should be kept in mind when assessing massive upper GI bleeding, as an unusual cause of bleeding gastric ulcer, given that bleeding is an uncommon presenting feature of GI lymphoma.

## 1 Introduction

Mantle cell lymphoma (MCL) is a mature B-cell non-Hodgkin lymphoma. Although it is often discussed alongside the clinically indolent types of non-Hodgkin lymphoma, its behavior is more often that of a disease that is aggressive.^[1]^ In the United States and Europe, MCL accounts for approximately 7% of adult non-Hodgkin lymphomas, with an annual incidence of 4 to 8 cases per million people.^[1]^ MCL may affect any part of the digestive tract, with lymphomatous intestinal polyposis as an occasional presentation.^[2]^ The stomach (57 percent), duodenum (52 percent), jejunum/ileum (87 percent), colon (90 percent), and rectum (69 percent) were all included in a prospective clinicopathological review of 31 cases of gastrointestinal tract involvement.^[3]^

Patients with gastric lymphoma have nonspecific symptoms similar to those seen in more common gastric conditions such as peptic ulcer disease, gastric adenocarcinoma, and non-ulcer dyspepsia. Occult gastrointestinal bleeding constitutes approximately 19 percent of the presenting symptoms of all gastric lymphomas.^[4]^

We present a case of mantle cell lymphoma that initially presented with an unusual massive upper gastrointestinal bleeding.

## 2 Case presentation

A 60-year-old previously healthy gentleman with a history of peptic ulcer disease was diagnosed four months prior to admission in his home country by endoscopy and treated medically with antibiotics as per the patient. He presented to our hospital with a few days’ history of recurrent episodes of black tarry stool, mild nausea and heartburn, but no vomiting or frank abdominal pain. The patient denied any history of alcohol consumption, painkillers, or herbals.

**Figure 1:**
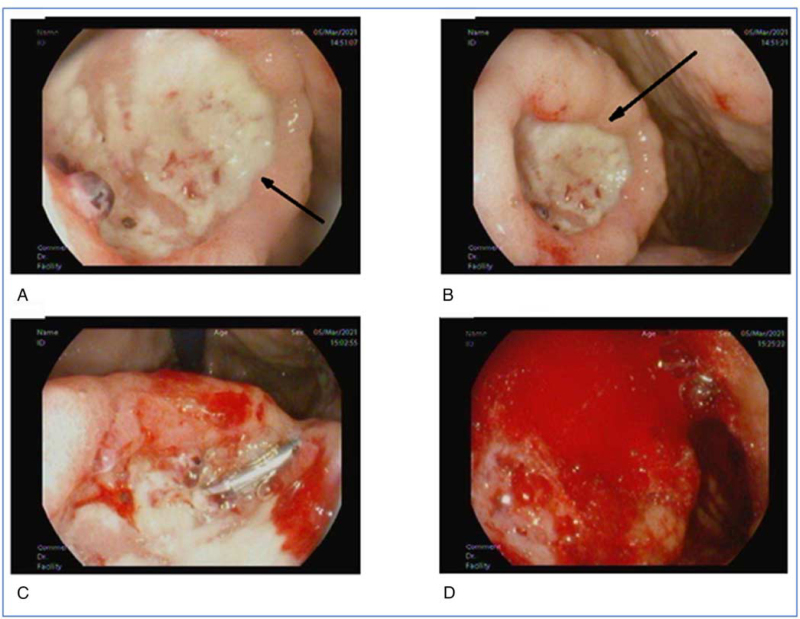
A, B: Large Rolled Edges Greater Curvature Ulcers. C, D: Bleeding during the attempt to deploy an endoclip.

On admission, his blood pressure was 115/67 mm Hg, and his heart rate was 115 beats per minute. Physical examination results were otherwise unremarkable. Initial blood test showed Hb 6.6 g/dl (Table 1). The patient was admitted for a short stay and underwent Esophagogastroduodenoscopy. Esophagogastroduodenoscopy revealed a large, rolled edges greater curvature ulcer measures around 3 × 2 cm, with an eccentric blood vessel (Fig. 1). The endoclip was deployed but the vessel started to ooze heavily thereafter. The patient was transferred to the intensive care unit for stabilization and monitoring.

**Table 1:** Laboratory tests upon admission.

Detail	Value w/Units	Normal Range
Beta 2 Microglobulin	2.21 mg/L	0.80–2.20
WBC	10.2 x10^3/uL	4.0–10.0
Hgb	6.3 gm/dL	13.0–17.0
MCV	87.0 fL	83.0–101.0
Platelet	233 x10^3/uL	150-400
INR	1.1	
APTT	21.9 seconds	24.6–31.2
Urea	17.0 mmol/L	2.5–7.8
Creatinine	68 umol/L	62-106
ALT	13 U/L	0-41
Retic #	144.8 x10^3/uL	50.0–100.0
Retic %	6.2%	0.5–2.5

ALT = alanine transaminase, APTT = activated partial thromboplastin time, Hgb = hemoglobin, INR = international normalized ratio, MCV = mean corpuscular volume, WBC = white blood cell.

The interventional radiology team was contacted for embolization. There was no contrast extravasation during computed tomography angiography by interventional radiology, so no embolization was done. Contrast-enhanced computed tomography abdomen showed features of gastric carcinoma with cervical peri gastric and possibly left para-aortic metastatic lymph nodes and suspected transverse mesocolon peritoneal nodules. During hospitalization, the patient developed multiple episodes of symptomatic anemia and melena.

He was treated conservatively with blood transfusion and started on Traneximic acid for 3 days. A biopsy revealed Mantle cell lymphoma. Therefore, the patient underwent urgent hemostatic radiotherapy. The patient received single fraction radiation therapy 4 Gy.

The bleeding was resolved with a significant improvement in the patient status, and he was transferred to the general ward for further monitoring. The patient refused to initiate the chemotherapy and insisted on going back to his home country

## 3 Histopathology

**Figure 2:**
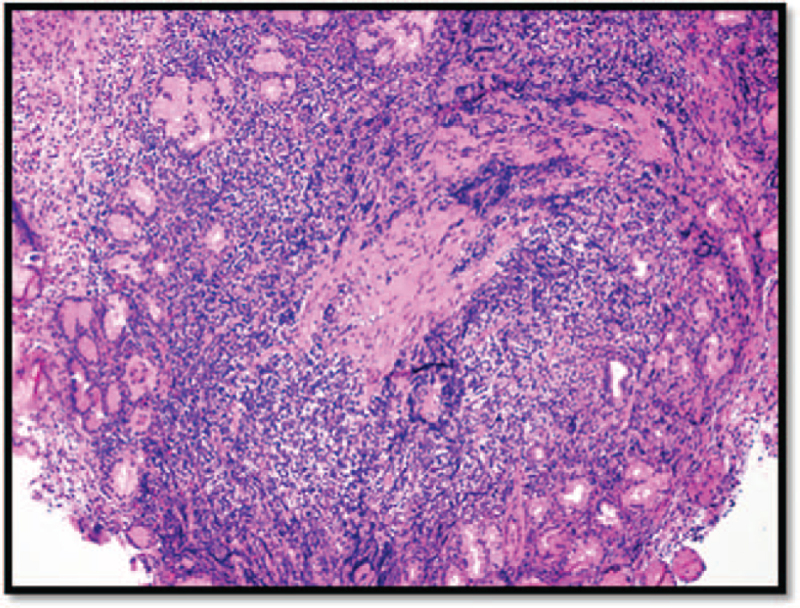
Low-magnification H&E image of the gastric biopsy. There is a dense lymphocytic infiltrate in the lamina propria. H&E = hematoxylin and eosin.

Sections from the gastric biopsy (Fig. 2) showed expansion of the lamina propria with sheets of small monotonous lymphocytes with small nuclei and perinuclear clearing artifacts.

No large cell or lymphoepithelial lesions were observed. Adjacent areas of ulceration and intestinal metaplasia were observed (not shown).

**Figure 3:**
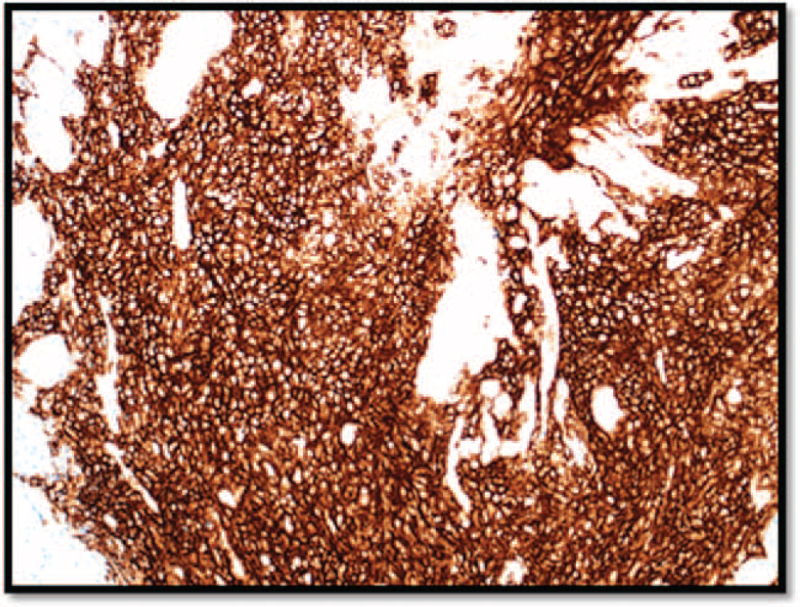
CD20 immunohistochemical staining was positive in neoplastic lymphocytes, confirming the nature of B cells. CD = the cluster of differentiation.

**Figure 4:**
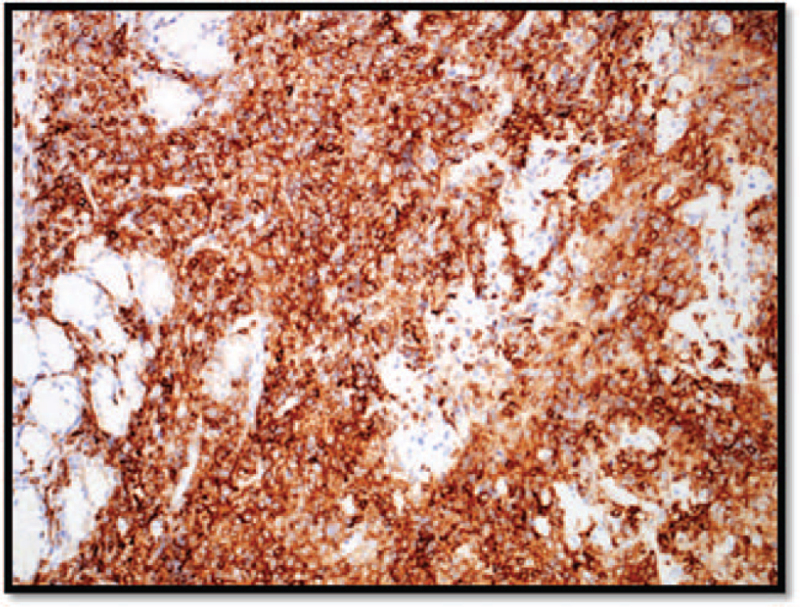
CD5 immunohistochemical staining is expressed in the non-neoplastic T cells in addition to the neoplastic B cells. CD = the cluster of differentiation.

**Figure 5:**
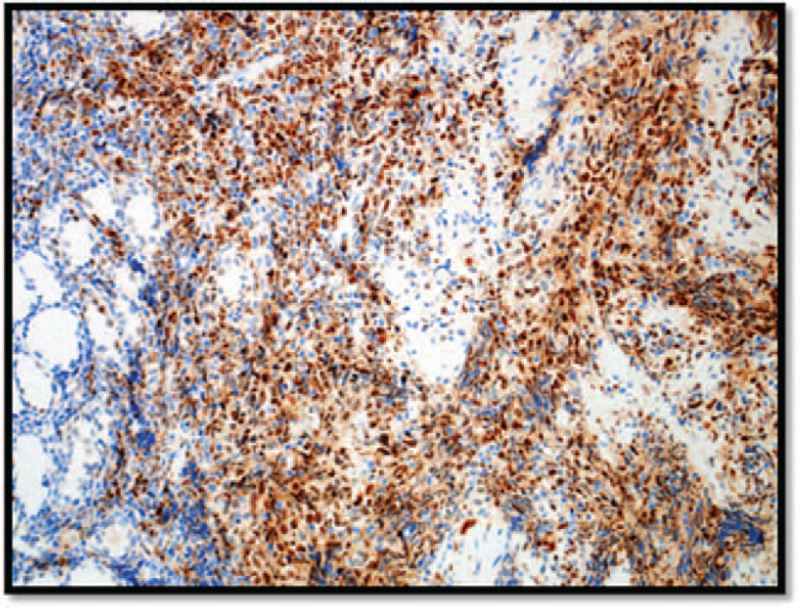
Cyclin-D1 immunohistochemical staining was expressed in neoplastic B cells confirming the diagnosis of mantle cell lymphoma. CD = the cluster of differentiation.

Immunohistochemical staining showed that the neoplastic cells were B-cell in nature, which stained positive for the cluster of differentiation (CD)20 (Fig. 3), and co-expressed CD5 (Fig. 4), cyclin-D1 (Fig. 5) and SOX-11 (not shown). The cells were also positive for BCL2 expression. The proliferation marker Ki-67 was positive in 20% of cells (not shown). Neoplastic cells were negative for CD10 and CD23 (not shown). Non-neoplastic T cells are highlighted with CD3 (not shown). The morphological and immunophenotypical features were consistent with those of mantle cell lymphoma. Bone marrow biopsy is hypercellular for age (∼45% to 65%), with active granulopoiesis, prominent erythropoiesis and increased megakaryocytes. The biopsy shows multiple small inter- and paratrabecular atypical lymphoid aggregates composed of small lymphoid cells comprising either B cells only or mostly B cells mixed with some T cells. The aggregates are also positive for PAX5, CD79, Cyclin D1, CD5, and strongly positive for Bcl2 while negative for Bcl6, CD23, and CD10.

Flow cytometry on the bone marrow aspirate shows a small population of monotypic B-cell (1%), expressing CD19, CD20, CD79, CD5, CD38, CD43, FMC7, IgD, and IgM with lambda light chain restriction. FISH analysis revealed a dual fusion indicating IGH/CCND1 XT rearrangement in 1.5% of the cells analyzed.

Overall findings are consistent with bone marrow involvement by Mantle cell lymphoma, which makes it a case of stage IV primary gastric Mantle cell lymphoma

## 4 Discussion

At the time of diagnosis, the majority of patients with MCL are in an advanced stage of the disease (70 percent). While lymphadenopathy is the primary symptom in around 75% of patients, extranodal disease is the primary clinical manifestation in the remaining 25%.^[5]^ The lymph nodes, spleen (45 to 60 percent), Waldeyer's ring, bone marrow (>60 percent), blood (13 to 77 percent), and extranodal sites like the gastrointestinal tract, breast, pleura, and orbit are all common sites of involvement.^[6]^ The explanation for mantle cell lymphoma's affinity for the gastrointestinal tract is unknown.^[6]^

Gastrointestinal MCL of a primary origin with no nodal or extranodal manifestations constitute only around 4 to 9 percent of all primary non-Hodgkin lymphomas.^[7]^ It usually presents as multiple lymphomatous polyposis. However, it was described as a single polyp, ulceration, and mucosal infiltration.^[3]^

On the other hand, it is relatively common for the gastrointestinal (GI) tract to be involved in MCL patients and the prevalence is around 15 to 30 percent, at the time of diagnosis or relapse.^[8]^ Moreover, 90 percent of patients who have MCL with no treatment were found to have GI involvement although most of them had no GI complains.^[9]^

Patients with mantle cell lymphoma that has spread to the gastrointestinal tract may be asymptomatic, or experience a variety of symptoms, including abdominal pain, obstruction, diarrhea, melena, and hematochezia. The ileocecal region is the most common site of gastrointestinal tract involvement, but any area from the stomach to the rectum could be affected. In a study published by Romaguera et al, up to 26% of patients had gastrointestinal symptoms at the time of diagnosis.^[6]^

Endoscopy can reveal MCL involvement in the gastrointestinal tract that is not visible on radiological imaging. During the staging process and during the follow-up period for MCL patients, endoscopic examinations are recommended.^[10]^ Moreover, chemotherapy for primary gastrointestinal mantle lymphomas might result in potentially fatal gastrointestinal bleeding. Much attention should be paid to the prevention and treatment of primary gastrointestinal lymphoma gastrointestinal bleeding while receiving chemotherapy.^[11]^

It is usually not easy for GI bleeding to be controlled by endoscopic maneuvers only and it commonly requires surgical or radiological interventions. One of the newly FDA approved interventions is the Hemospray that begin hemostatis in risky bleeding cases, which provide more time for definitive treatment.^[12]^

The chemotherapy regimen for MCL is mainly a combination regimen of CVP (cyclophosphamide, vincristine, prednisone) and CHOP (cyclophosphamide, hydroxydaunomycin, Oncovin [vincristine], prednisone) regimen, hyper-CVAD (hyperfractionated cyclophosphamide, vincristine, doxorubicin [adriamycin], dexamethasone, methotrexate, and cytarabine) with or without rituximab, hyper-CVAD with autologous stem cell transplantation, R-CHOP (CHOP plus rituximab), and single alkylating agents like chlorambucil.^[13]^

For relapses and refractory cases, Bortezomib, Lenalidomide, and Ibrutinib are used. The prognosis is poor with ten years survival of only around 5% to 10% of patients, and median survival about 3 years.^[13]^

## 5 Conclusions

MCL should be kept in mind when assessing massive upper GI bleeding, as an unusual cause of bleeding gastric ulcer. Also, predict the advancement of bleeding or onset of new bleeding after initiation of chemotherapy in patients with MCL.

## Acknowledgments

The authors would like to thank the patient for allowing them to share his details with the medical community. Additionally, they acknowledge the Qatar National Library for funding the open access fees of this publication.

## Author contributions

**Conceptualization:** Khaled Ali, Mhd Baraa Habib.

**Visualization:** Ferial Alloush.

**Writing – original draft:** Ferial Alloush, Khaled Ali.

**Writing – review & editing:** Mohamed Yassin.
